# HSV: The scout and assault for digestive system tumors

**DOI:** 10.3389/fmolb.2023.1142498

**Published:** 2023-02-28

**Authors:** Sheng Li, Qingbo Li, Yi Ren, Jia Yi, Jinhe Guo, Xianbin Kong

**Affiliations:** College of Traditional Chinese medicine, Tianjin University of Traditional Chinese Medicine, Tianjin, China

**Keywords:** HSV, digestive system tumors, oncolytic virus, immunology, cancer

## Abstract

More than 25% of all malignant tumors are digestive system tumors (DSTs), which mostly include esophageal cancer, gastric cancer, pancreatic cancer, liver cancer, gallbladder cancer and cholangiocarcinoma, and colorectal cancer. DSTs have emerged as one of the prominent reasons of morbidity and death in many nations and areas around the world, posing a serious threat to human life and health. General treatments such as radiotherapy, chemotherapy, and surgical resection can poorly cure the patients and have a bad prognosis. A type of immunotherapy known as oncolytic virus therapy, have recently shown extraordinary anti-tumor effectiveness. One of the viruses that has been the subject of the greatest research in this field, the herpes simplex virus (HSV), has shown excellent potential in DSTs. With a discussion of HSV-1 based on recent studies, we outline the therapeutic effects of HSV on a number of DSTs in this review. Additionally, the critical function of HSV in the detection of cancers is discussed, and some HSV future possibilities are shown.

## 1 Introduction

Digestive system tumors (DSTs) are the most common cancers worldwide (Sung et al., 2021). The following DSTs have the highest global mortality rates in 2020: esophageal (5.5%), gastric (7.7%), pancreatic (4.7%), liver (8.3%), gallbladder (0.9%), and colorectal (9.4%). Except for gallbladder cancer, the rest of them increased in comparison to 2018 statistics (Bray et al., 2018). It is undeniably a major threat to human health and an urgent issue for both researchers and healthcare workers to address.

Currently, the most effective treatment modalities are still surgical resection, chemotherapy, radiotherapy, and conventional anti-cancer drug therapy, either alone or in combination (Zhao and Liu, 2020; Wei and Hackert, 2021). The prognosis of these treatments, however, has never met expectations, according to numerous reports (Lu et al., 2021; Yang et al., 2021), and patient survival rates after 5 years have not significantly increased. More reversing interventions need to be investigated and promoted in order to change this phenomenon as soon as possible. Many researchers have been successful in developing effective therapeutic targets (Li et al., 2022a; Xu et al., 2022) and microbial studies in recent years, and are moving toward targeted therapies and immunotherapies that can kill tumor cells more precisely (McAllister et al., 2019; Lu et al., 2021). Meanwhile, cancer patients have received immunotherapies like immune checkpoint therapy (ICT), tumor vaccines, immune adoptive therapy, and immunomodulator (Chen et al., 2021). These efforts all contribute to the search for distinctive breakthroughs against digestive tumors.

The oncolytic virus (OV) is a recent cancer treatment method among immunotherapies (Li et al., 2022d). OV selectively targets tumor cells as hosts, activates innate as well as adaptive anti-tumor immunity, and productively lyses tumor cells (Melcher et al., 2021; Li et al., 2022b). On the basis of the above advantages, OV gradually evolves into a potentially promising alternative and curative treatment to traditional cancer therapy. Oncolytic viruses come in a variety of forms, including those involving DNA, single-stranded RNA (ssRNA), and double-stranded RNA (dsRNA) viruses. Reports of associated clinical trials are rapidly rising (Fundora Ramos et al., 2021; Huang et al., 2021; Li et al., 2022c; Li and Sun, 2022). In 1991, herpes simplex virus (HSV) became the first genetically engineered virus to be utilized as an oncolytic virus (Martuza et al., 1991). T-VEC, a type of oncolytic virus that expresses granulocyte-macrophage colony-stimulating factor (GM-CSF), was approved by the FDA 25 years later (Pol et al., 2016). It is undoubtedly a milestone in viro-immunotherapy and believed that to be bloom in the future. Recent progress in viro-immunotherapy has reached this point, and additional growth is anticipated. In this review, we summarize the role of HSV and the diagnostic value that exists in the treatment of DSTs, as well as the clinical value and future of HSV.

## 2 Oncolytic virus

Tumor cells are granted several “privileges” in order to facilitate malignant proliferation and immune escape, but this comes at the expense of disrupting regulatory pathways and suppressing immune gene expression and inheritance (Ilkow et al., 2014). Oncolytic viruses are typically genetically modified viruses based on natural viruses that exploit this vulnerability to penetrate the barrier, invade cancer cells, and are greatly supported by the cancer cells’ crippled, malignant metabolic base (Hanahan and Weinberg, 2011), which serves as the foundation of an oncolytic virus as a new anti-tumor strategy. OV has been the subject of extensive investigation for more than 70 years. Despite its long-reported efficacy (Moore, 1949a;Moore, 1949b;Nemunaitis, 1999), the FDA just first approved T-VEC in 2015 as an oncolytic agent for the treatment of metastatic melanoma. T-VEC was produced on the basis of HSV-1 with the insertion of the gene called GM-CSF (Andtbacka et al., 2015; Pol et al., 2016).

The anti-tumor mechanism of oncolytic virus has been interpreted as: 1) selective infiltration and replication of tumor cells as hosts, causing direct cell lysis and death; and 2) induction of a systemic anti-tumor immune response, reactivating dormant innate and adaptive immunity (Russell et al., 2012; Engeland, 2020), restoring “control” to established immune escapees (Kaufman et al., 2015). However, we should mention that the oncolytic virus’ rebuilding of the tumor microenvironment (TME) has far-reaching implications for the latter (Zhu et al., 2022b; Su et al., 2022). These mechanisms have been thoroughly investigated, providing us with an insight into the irreplaceable nature of the oncolytic virus (Lichty et al., 2014; Heidbuechel and Engeland, 2021).

## 3 HSV as the oncolytic virus

### 3.1 Structure and advantages of HSV

The herpesvirus family is a sizable group of a large amount of double-stranded DNA viruses with envelopes (Scanlan et al., 2022). It was split up into the α, β, and γ subfamilies in 1978 based on the biological characteristics and genetic makeup of the viruses. HSV is a representative species of the α subfamily of herpesviruses, and in contrast to the other two, the α subfamily of herpesviruses is unique in having neuroinvasiveness (Knipe et al., 2013). HSV is split into the HSV-1 and HSV-2 serotypes depending on the antigenicity of each. The two virus types share 50% of their DNA, and they both carry common antigens between them as well as type-specific antigens. The viruses are round in shape and have 11 glycoproteins on their envelope surface that, when changed, have been proven to improve targeting to cancer cells (Sokolowski et al., 2015). Another factor in the choice of HSV as an oncolytic virus vector, in relation to this selective invasive effect, is that patients can still be kept safe with powerful anti-herpesviral medications like ganciclovir (GCV), even in life-threatening situations brought on by dose or self-specific reactions (Hartkopf et al., 2011; Koch et al., 2020).

**FIGURE 1:**
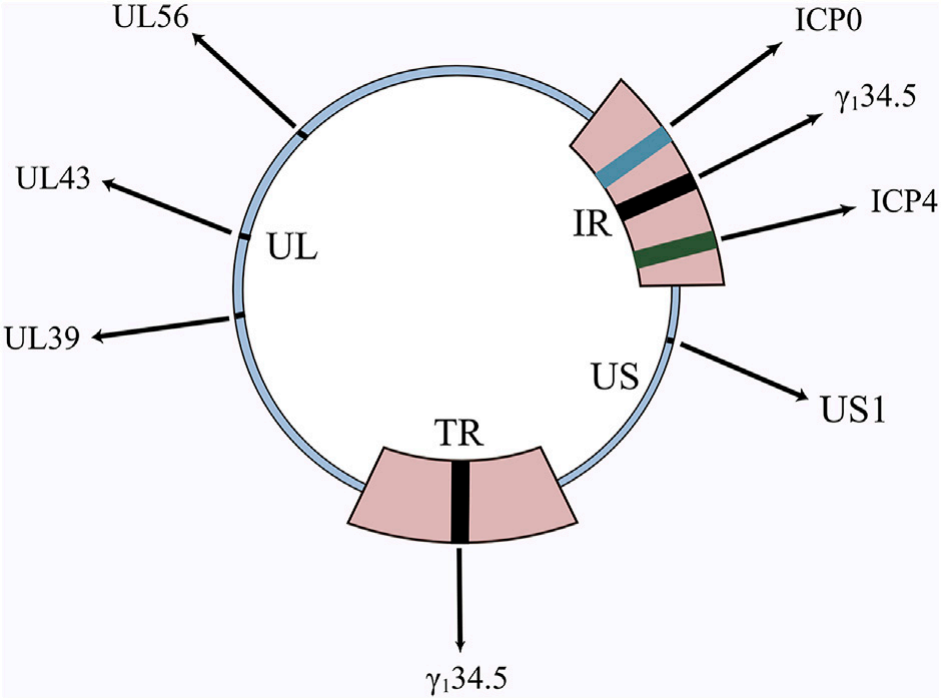
Schematic diagram of HSV genome and important genes. γ_1_34.5, ICP0, ICP4, UL39, UL43, UL56 are all important genes in the HSV genome. The deletion of these genes, as well as the insertion of numerous other genes, has resulted in the birth of a diverse family of oncolytic HSVs.

HSV-1 has a genome of 150 kb and HSV-2 has a genome of 154 kb (Watson et al., 2012), both consisting of covalently linked unique long (UL) and unique short (US) fragments, respectively bordered by inverted repetitions (TR and IR) **(** Figure 1 **)**. Nearly half of the HSV genome is composed of non-essential genes, with a large scope for genetic modification, allowing both easy modification and the simultaneous insertion of large segments of exogenous genes or multiple transgenes into the viral genome (Table 1). This provides a great convenience for HSV to be used as an oncolytic virus. Other viruses only bind to one receptor, while HSV has four cellar receptors. HSV can infect practically all cells thanks to this advantageous characteristic (Watanabe and Goshima, 2018). Because of this, when HSV is used as an oncolytic agent, the problem of treatment resistance is considerably diminished. The aforementioned proof is the main justification for academics’ intense interest in oncolytic HSV.

**TABLE 1:** A summary of genetic modifications and experimental profiles of oncolytic HSVs.

Name	Type	Genetic Modifications	Experiment Types	Target DSTs	Positive/Negative	References
G207	HSV-1	Deletion of two copies of γ134.5, inactivation of UL39	Clinical trials (NCT03911388, NCT04482933, NCT02457845, NCT00157703, NCT00028158); *in vivo*; *in vitro*	Gastric cancer; colorectal cancer	Positive	(Bennett et al., 2002; Stanziale et al., 2002)
G47Δ	HSV-1	Deletion of α47based onG207	*in vivo*; *in vitro*	Esophageal cancer; gastric cancer	Positive	(Sugawara et al., 2020; Yajima et al., 2021)
NV1020	HSV-1	Deletion of UL56, one copy of ICP0, ICP4 and γ134.5, insertion of a fragment of HSV-2 DNA encoding several glycoprotein genes	Clinical trials (NCT00149396, NCT00012155); *in vivo*; *in vitro*	Gastric cancer; colorectal cancer	Positive	(Bennett et al., 2002; Kemeny et al., 2006; Fong et al., 2009)
NV1066	HSV-1	Deletion of one copy of ICP0, ICP4 and γ134.5, insertion of EGFP	*In vivo*; *in vitro*	Gastric cancer; pancreatic Cancer	Positive	(Eisenberg et al., 2010)
hrR3	HSV-1	Inactivation of UL39	*In vivo*; *in vitro*	Colorectal cancer	Positive	(Yoon et al., 1998)
OH2	HSV-2	Deletion ofICP47 and two copies of γ134.5, insertion of GM-CSF	Clinical trials (NCT05248789, NCT05232136, NCT05698459, NCT05235074, NCT04637698, NCT03866525, NCT04616443, NCT04386967, NCT05648006); *in vivo*	Esophageal cancer; gastric cancer; cholangiocarcinoma; colorectal cancer	Positive	(Zhang et al., 2021)
HF10	HSV-1	Deletion of UL43, UL49.5, UL55, UL56 and LAT	Clinical trials (NCT03153085, NCT03259425, NCT02428036, NCT03252808, NCT02272855, NCT01017185); *in vivo*; *in vitro*	Pancreatic Cancer	Positive	(Kasuya et al., 2014; Yamamura et al., 2014)
Cgal-Luc	HSV-1	Insertion of the luciferase gene based on HSV-1 17syn+	*In vivo*; *in vitro*	Hepatocellular carcinoma	Positive	(Argnani et al., 2011)
H6-Luc	HSV-1	Insertion of the luciferase gene based on HSV-1 HFEM	*In vivo*; *in vitro*	Hepatocellular carcinoma	Positive	(Argnani et al., 2011)
LCSOV	HSV-1	Linking essential viral glycoprotein H gene with the liver-specific apolipoprotein E (apoE)-AAT promoter, insertion of complementary sequences from miR-122a, miR-124a, and let-7	*In vivo*; *in vitro*	Hepatocellular carcinoma	Positive	(Fu et al., 2012)
Ld0-GFP	HSV-1	Insertion of GFP based on an ICP0-Null HSV-1	*In vitro*	Hepatocellular carcinoma	Positive	(Luo et al., 2019)
NV1023	HSV-1	Insertion of a fragment of HSV-2 DNA containing the genes US2-2 to US2-5 based on NV1020	*In vitro*	cholangiocarcinoma	Positive	(Jarnagin et al., 2006)
VG161	HSV-1	Deletion of two copies of γ134.5, insertion of IL-12, IL-15, IL15RA and PL-L1b (TF-Fc peptide)	Clinical trials (NCT05223816, NCT05223816, NCT04758897, NCT04806464); *in vivo*; *in vitro*	Colorectal cancer	Positive	(Chouljenko et al., 2020)
HSV1716Ing4	HSV-1	Deletion of two copies of γ134.5, insertion of Ing4	*In vitro*	Colorectal cancer	Positive	(Conner and Braidwood, 2012)
HSV-HMGB1	HSV-1	Deletion of two copies of γ134.5, insertion of HMGB1	*In vitro*	Colorectal cancer	Positive	(Shayan et al., 2022)

### 3.2 How does HSV work?

#### 3.2.1 Direct invasion and lysis of cancer cells

Pattern recognition receptors (PRR) consisting of Toll-like receptors (TLR) (Bansode et al., 2021), NOD-like receptors (NLR) (Dutta et al., 2015), and RIG-I-like receptors (RLR) (Unterholzner, 2013) that activate antiviral mechanisms are present on the surface and in the cytoplasm of normal cells. When a viral infection is encountered, PRR performs its vital function of antigen recognition and immune initiation at this moment. Immune signals are transmitted through a variety of different pathways, leading to the synthesis and release of a number of inflammatory factors, such as type I interferon (IFN-I) (Lin and Zheng, 2019; Zhu et al., 2022a; Li et al., 2022e; Gong et al., 2022).

The ingenious fact is that HSV is skilled at finding weaknesses in cancer cells’ faulty antiviral response system. It initially infects cancer cells by identifying and attaching to the proper receptors and then begins reproduction and amplification (Kaufman et al., 2015). For instance, HSV-1 enters the cell with the help of HVEM (herpesvirus entry medium) or envelope glycoproteins (Bommareddy et al., 2017). After DNA is inserted into the nucleus of cellular tumor cells, HSV uses intranuclear RNA polymerase to transcribe its own DNA into mRNA and then uses intracellular organelles to direct the synthesis of some proteins. These proteins, usually DNA replication enzymes and regulatory proteins, are synthesized preferentially at the earliest stage (Rice et al., 1995). This is followed by the replication of viral DNA and finally the synthesis of structural proteins such as capsid proteins and envelope proteins (Cui et al., 2022). The assembly of progeny virions is done in the nucleus (Mohan et al., 2020).

**FIGURE 2:**
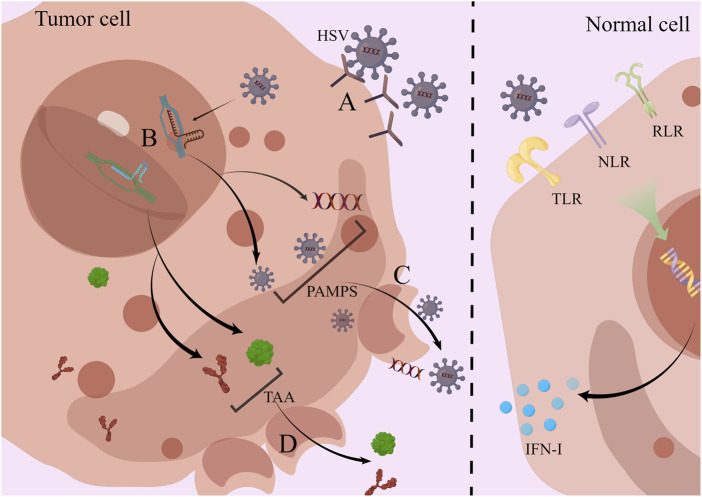
HSV directly infects and lyses tumor cells; normal cells activate antiviral effects *via* PRR. **(A)** HSV enters cells *via* HVEM or glycoproteins in the envelope. **(B)** Inside the nucleus, HSV uses cells for self-replication and value-added, producing large amounts of viral nucleic acids, viral capsid proteins, and progeny viral particles. **(C)** Viral proliferation leads to cell lysis, and PAMPS are released into the outside of the cell. **(D)** Along with cell lysis, TAA is also released which is highly expressed in tumor cells. In contrast to tumor cells, normal cells rely on PRRs such as TLR, NLR, and RLR to activate antiviral mechanisms and secrete inflammatory factors such as type I IFN to kill the virus.

Continuous proliferation depletes tumor cells of resources and triggers its lysis apoptosis. Viral nucleic acids, capsid proteins, and new progeny virions during proliferation (Wang et al., 2022a), are released as the cancer cells are lysed and recognized as pathogen-associated molecular patterns (PAMPS). Cancer cell tumor-associated antigens (TAA) and damage-associated molecular patterns (DAMPS) have also been made public (Kaufman et al., 2015) **(** Figure 2 **)**.

#### 3.2.2 Remodeling the tumor microenvironment and inducing systemic immunity

Typically, solid tumors contain a range of cellular and non-cellular components, consisting of immune cells, fibroblasts, and endothelial cells of the vascular system, cancer-associated fibroblasts (CAFs), extracellular matrix (ECM), in addition to cancer cells. These components make up the TME (Belli et al., 2018). Cancer cells and TME interact to create a sophisticated immune evasion network (Hanahan and Coussens, 2012), and this “fortress” has a negative impact on the body’s anti-tumor response (Gujar et al., 2018). It is crucial to recognize that TME creates a barrier or trap for the oncolytic virus (Vähä-Koskela and Hinkkanen, 2014), and the disturbance of capture or rejection is frequently what makes intratumoral injection superior to other delivery methods (Osali et al., 2020).

HSV remodels a “cold tumor” in an immunosuppressed state into a “hot tumor” in an immune-activated state. The distinction between “cold” and “hot” is made from an immunological perspective. T-cell are unable to infiltrate cold tumors, whereas hot tumors exhibit significant molecular characteristics of immune activation and an abundance of infiltrating T-cell (van der Woude et al., 2017). Infiltrating by T-cell, especially CD8^+^ T-cell, has been shown by several pieces of evidence to correlate with increased cancer cell killing and patient survival (Galon and Bruni, 2019; Newman et al., 2020). The change from cold to hot tumors can lead to an enhanced effect of TME receiving immune recognition, and inflammatory and immune cells are able to infiltrate toxicity into the TME to kill cancer cells (Samson et al., 2018). Moreover, this reversal of tumor induced by HSV has a distal effect and is not only present in certain specific sites or systems. Further evidence suggests that remodeling of TME by HSV with arousal of effector cells is a systemic immune activation response. For instance, in a mouse experiment, both the T-VEC injected area and the contralateral non-injected area showed morphological shrinkage of the tumor, and a significant rise in CD8^+^ T-cell was observed (Moesta et al., 2017). Against tumors, these T-cell are unquestionably effective. Clinical trial outcomes are also in agreement with this. Two lines of evidence demonstrating that cytotoxic T-cell produced after intra-tumor injection of T-VEC can be found in peripheral blood further support the general consensus that they induce systemic antitumor immunity (Kaufman et al., 2010; Ribas et al., 2017). In addition to CD8^+^ T-cell, variations of Treg cell levels occupy an essential place in the intricate network of TME. In human and mouse tumors, Treg cells foster tumor immune escape of cancer cells (Hori et al., 2003; Rech et al., 2012). A decrease of its level within the tumor has also been proved to be connected with the intervention of oncolytic viruses. In a mouse melanoma model, injection of oncolytic HSV leads to a decrease of intratumoral Tregs (Bozhanova et al., 2022). However, it has also been indicated that treatment with oncolytic HSV leads to an increase rather than a decrease in intratumoral Tregs levels (Ring et al., 2017). The possible explanation for the opposite result may be that oncolytic HSV treatment alone is not adequate to generate a transformation-mimetic advantage in some tumors prone to Tregs infiltration.

**FIGURE 3:**
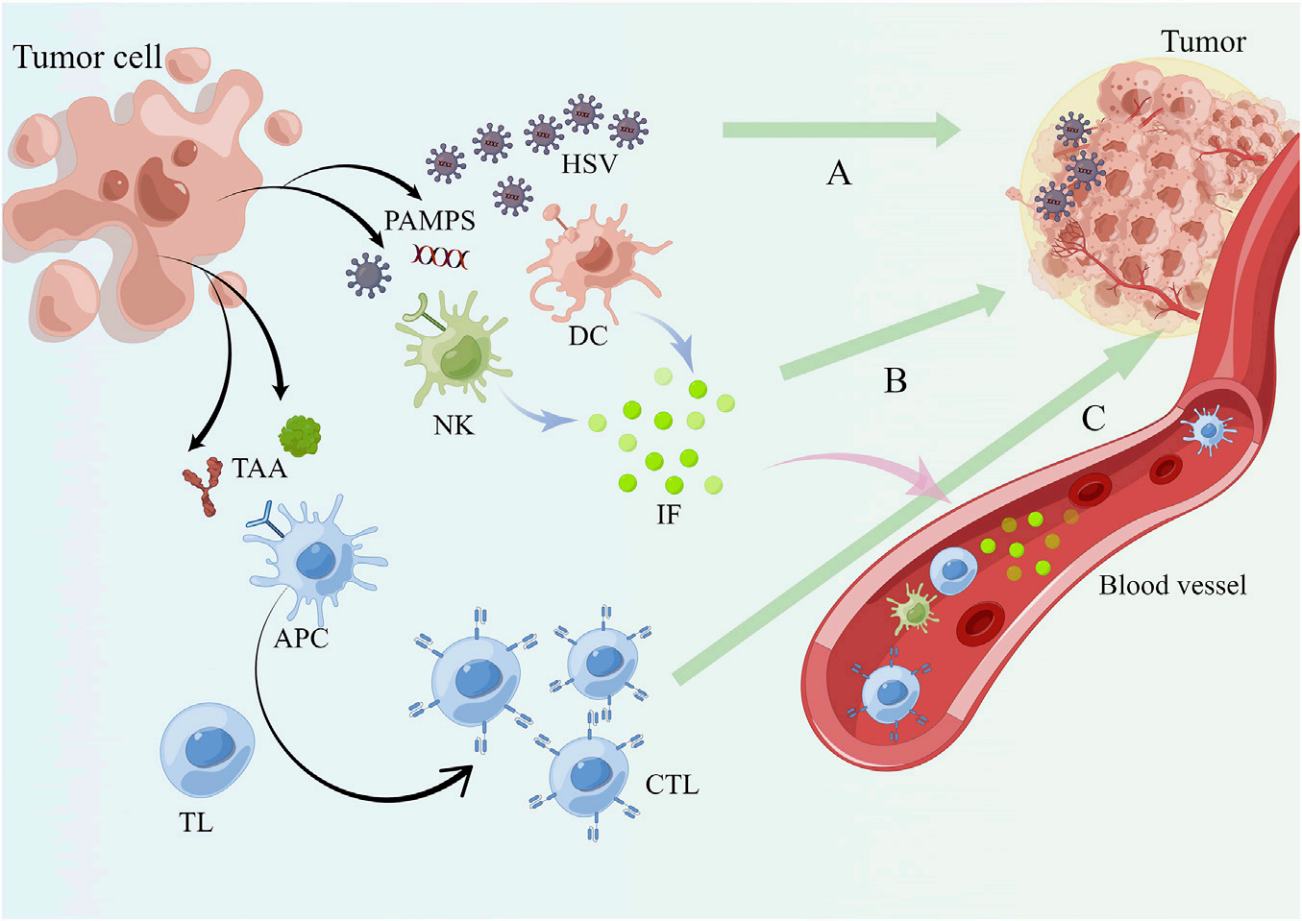
HSV remodels the TME and induces systemic anti-tumor immunity. **(A)** After the tumor cells are lysed, PAMPS are released and the mature HSV virus continues to infect other tumor cells in the TME. **(B)** PAMPS are recognized by immune cell subsets present in the TME such as DC, NK and APC relying on PRR which secrete immune inflammatory factors like IFN-γ, IL-6 and IL-12. **(C)** APC phagocytose TAA and PAMPS and present antigens to T lymphocytes, activating them to become cytotoxic T lymphocytes to complete the adaptive immunity against tumor cells. Blood circulation allows mature progeny viruses, immune cells and immune molecules to reach the whole body and a systemic anti-tumor immune response occurs.

When released into the tumor and the circulation, PAMPS is recognized by PRR of various immune cell subsets, consisting of dendritic cells (DCs), natural killer (NK) cells, antigen presenting cells (APCs), and the corresponding immune cells secrete inflammatory factors such as IL-6, IL-12, and IFN-γ(Yan and Chen, 2012). As more immune cells get enriched around the tumor tissue as a result of these successive events (Oosenbrug et al., 2017), more these important immunoreactive substances will work on both the tumor cells and the HSV progeny viruses themselves, causing immunogenic cell death (ICD) (Kroemer et al., 2013). Usually the ICD involves immunogenic apoptosis, necrosis, pyroptosis, and autophagic cell death (Guo et al., 2014). Each of these cell death patterns can be instrumental in the effect of antitumor immune response (Workenhe et al., 2014). High-mobility group box 1 (HMGB1) is an intracellularly derived DAMPS that serves as a sensitive inflammatory signal and functions as a warning for cell death and microbial invasion (Yanai et al., 2009). Its release was examined in several oncolytic HSVs infestations (Workenhe et al., 2014). In this experiment, the rate of apoptosis was remarkably accelerated in all cases, indicating that ICD associated with HMGB1-mediated is quite beneficial for inducing antitumor immunity. The promotion of the immune process by ICD as resulted from other modalities has been equally reported (Workenhe and Mossman, 2014). Tumor cells struggle to easily escape and are subjected to several anticancer immunoreactive chemicals, whereas TAA is collected by APCs and given to T-cell in a cross-presentation pattern, activating their adaptive immune responses against that antigen (Shi et al., 2020) **(** Figure 3 **)**. The easy entry of HSV into cells and the necessity for the activation of immune cells and the promotion of the release of inflammatory factors are both mediated by signaling pathways associated with viral clearance, such as TLR and protein kinase RNA activation (PKR) pathways, which are restricted in cancer cells (Zhao et al., 2020).

## 4 Diagnosis and treatment of DST by HSV

### 4.1 Esophageal cancer

According to histopathology, esophageal cancer is classified into two types: squamous cell carcinoma (ESCC) and adenocarcinoma (EAC) (Siewert and Ott, 2007). EAC is more common in North America as well as Western Europe, whereas ESCC is more common in Eastern Europe and Asia (Ajani et al., 2019). However, conventional treatments frequently have poor outcomes, and resistance to chemotherapy and radiotherapy in both cases has resulted in the development of new therapies (Zhao et al., 2019). Oncolytic virus therapy has been a standout among emerging immunotherapies.

The novel telomerase-specific OV (OBP-301) in combination with locally focused irradiation was effective and tolerated in older ESCC patients, according to the findings of a phase I/II study (Tanabe et al., 2015). G47Δ has been identified as a highly expected innovative cancer therapeutic agent as a third-generation lysogenic HSV-1. Its α47 gene was removed when compared to G207 (prior generation HSV-1). G47Δ demonstrated a strong ability for replication and pro-apoptotic effects in each of the eight human esophageal cancer cell lines examined *in vitro*. This evidence unquestionably highlights the safety of G47Δ because it did not impair mouse survival whether either topically or orally for 2 months (Yajima et al., 2021). The tegument protein VP22, which acts as a heterologous protein transporter across the host cell membrane following HSV-1 infiltration of cancer cells, has been reported to improve the antitumor activity of the ESCC cell line Eca109 *in vitro* and *in vivo* by mediating the intercellular delivery of the phosphatase and tensin homolog (PTEN) protein. Surprisingly, VP22 does not inhibit tumor growth or anti-angiogenesis on its own. It must rely on intercellular delivery of PTEN, a tumor suppressor, to increase its concentration before it becomes effective. The gap in HSV treatment of EAC remains unfilled, and we anticipate that the value of HSV will be realized in this field.

### 4.2 Gastric cancer

Less than 25% of people with gastric cancer survive it for 5 years (Gamboa and Winer, 2019) and is prone to peritoneal metastases (Macrì and Morabito, 2019; Manzanedo et al., 2019), which can lead to peritoneal cancer at diagnosis and following surgery. Due to their link, the two have been the subject of much parallel research. The oncolytic activity of the two oncolytic HSVs, G207 and NV1020, is inconsistent since the different deletion of γ_1_34.5 and ICP6 (ribonucleotide reductase) genes (Ma et al., 2018; Friedman et al., 2021). They quickly infect human gastric cancer cells *in vitro* and kill them. When given intraperitoneally at large doses, cytotoxicity was also noted in a created animal model of peritoneal disseminated gastric cancer. The outcome, however, does not bode well for intravenous administration methods (Bennett et al., 2002). This highlights the value of localized care. The human gastric cancer cell line OCUM shown sensitivity to NV1066 in both *in vitro* and *in vivo* disseminated peritoneal cancer models, with strong cytotoxicity detected. NV1066 is an attenuated HSV-1 that possesses an enhanced green fluorescent protein (EGFP) gene and can be induced to express EGFP when infecting tumor cells. Additionally, EGFP expression was seen in the lesions (Stanziale et al., 2004). This finding lends support to the use of HSV as a means of lesion localization and therapeutic agent for peritoneal or gastric cancer.

Patients with stomach cancer experience symptoms that range from mildly uncomfortable to transient dyspeptic symptoms in the early stages of the disease to severe symptoms such as malignant gastric hemorrhage, perforation, and blockage in the late stages (Wang et al., 2022a). Third-generation oncolytic HSV-1 showed great killing potential in an *in vitro* cell infection assay, pushing 50% of gastric cancer cells over the survival limit in a short amount of time, according to an earlier investigation of oncolytic HSV killing gastric cancer cells (Wong et al., 2010). The combination of bevacizumab (a tumor angiogenesis inhibitor) and hrR3 (an oncolytic HSV) was only effective in an *in vivo* gastric cancer model (Deguchi et al., 2012), and the reasons for the lack of efficacy *in vitro* cell infection assays are still unknown. Platelet response factor-1 (TSP-1) was armed with a third-generation oncolytic HSV-1 vector, and its active expression was increased *in vivo* and *in vitro* compared to an unarmed one by viral vector replication (Tsuji et al., 2013). Another study found that arming increased antitumor efficacy (Matsumura et al., 2021), with the difference that the third-generation oncolytic HSV-1 vector was armed with suppressor of cytokine signaling 3 (SOCS3). SOCS3 was found to be silenced in a variety of cancer cells. These positive findings show how valuable HSV is as a “fighter jet carrier” laden with “aircraft.” The third-generation oncolytic HSV-1 G47Δ, which was previously discussed, has a significant therapeutic potential for the treatment of gastric cancer. Viral replication and quick cell death were seen in every of the 9 cell lines of human gastric cancer examined. Additionally, an *in vivo* G47 inoculation assay revealed a substantial inhibition of advanced gastric cancer and scirrhous-type gastric cancer (Sugawara et al., 2020).

The human telomerase reverse transcriptase promoter (T-hTERT) was regulated in investigations of the fourth-generation HSV carrying the ICP6 gene, resulting in improved expression of this gene, which is crucial for viral replication, and superior tumor suppression compared to the third-generation HSV-1 (Kato et al., 2021). Based on the genetic modification of HSV-2 HG52 that enables the virus to preferentially replicate in tumors and express GM-CSF, a phase I/II study was created to examine the safety and effectiveness of OH_2_ either alone or in conjunction with the anti-PD-1 antibody HX008 in patients. All the patients have malignant solid tumors, such as gastrointestinal cancers. The trial is currently ongoing, and the outcomes are unknown (NCT03866525). However, we can infer from this information that more and more modified viruses are being researched, which, by releasing more antigens, strengthen the immune response against cancers.

### 4.3 Pancreatic cancer

A highly aggressive tumor of the digestive system, pancreatic cancer is extremely aggressive and has the potential to spread (Zhang et al., 2022b). Its metastasis is intimately linked to circulating tumor cells (CTCs) and tracking CTCs enable earlier tumor recurrence risk prediction than is possible with traditional imaging techniques. Numerous studies have demonstrated that CTC identification will aid in the monitoring of recurring metastases, assessment of the prognosis of the patient, and direction of postoperative adjuvant therapy (Hodgkinson et al., 2014).

Recent evidence suggests that HSV has a non-negligible ability to act as a diagnostic agent for cancer dissemination status. By using a cell-surface marker-free, HSV-1-based technique, escaping CTCs from lesions that are going to “seed” and “colonize” elsewhere from blood samples can be detected (Zhang et al., 2016). This is related to the HSV’s ability to replicate only in tumor cells at the source. The importance of this research lies in its capacity to render these cancerous tumor cells incapable of hiding, providing medical personnel with an invaluable opportunity to stop and cure them. Oncolytic HSV can act as a “scout” and a highly accurate diagnostic tool for the rapid identification of pancreatic cancer micrometastases (Kelly et al., 2016). It is noteworthy that HSV excels in diagnostic procedures and can enhance a patient’s therapy options for malignancies, including pancreatic cancer.

Surely, we cannot dismiss the capability of HSV itself as an OV to kill tumor cells. The good therapeutic effects of this medication have been demonstrated by pathology reports from 17 patients (8 with pancreatic cancer) in a phase I dose-escalation clinical trial (Kasuya et al., 2014) after treatment with HF10 (a mutation of HSV-1). Two human pancreatic cancer cell lines, BxPC-3 and PANC-1, have shown inconsistent cytotoxicity in response to HF10 treatment either alone or in combination with erlotinib (Yamamura et al., 2014). The former showed the strongest cytotoxicity after combination, but the latter did not exhibit this combination effect, indicating that such medicines have a certain tendency to be selective towards cancer cells.

Except for the medication combination, the combination of hyperthermia and elevation of Hsp72 expression also increased the oncolytic impact of NV1066 (Eisenberg et al., 2010), resulting in a more potent death of pancreatic cancer cells. When HSV was employed in conjunction with chemotherapy and radiotherapy to treat pancreatic cancer, we discovered that the results were reversed. When pancreatic cancer cells were exposed *in vitro* to the chemotherapeutic agents 5-FU, CPT-11, or MTX, they significantly inhibited HSV-1 replication and cell dissolution (Dai et al., 2010). However, ionizing radiation (IR) exhibited a synergistic oncolytic effect with HSV-1 (Kulu et al., 2013). This suggests that we should not assume that combining two or more therapies will result in stronger tumor lysis, and that more research is needed to confirm the contradiction between the two.

### 4.4 Liver cancer

There are two types of liver cancer: primary liver cancer and secondary liver cancer. Hepatocellular carcinoma (HCC), intrahepatic cholangiocarcinoma, and mixed carcinoma are the three types of primary liver cancer. Mixed carcinoma is defined as having elements of both hepatocellular carcinoma and cholangiocarcinoma cell carcinoma. Secondary hepatocellular carcinoma develops when tumor cells from other sites spread to the liver *via* blood and colonize. Liver metastasis from colorectal cancer, liver metastasis from gastric cancer, liver metastasis from lung cancer, and liver metastasis from gynecological tumors like ovarian cancer and uterine cancer are all common. These are all referred to as liver metastases. Immunotherapy has also been adopted to treat liver cancer.

RANTES, B7.1, and GM-CSF are all immunostimulatory proteins. A single agent tumor vaccine secreting GM-CS or a multi-drug combination tumor vaccine secreting RANTES/B7.1/GM-CSF can effectively inhibit cancer cells and exert excellent anti-tumor effects when produced by gene transfer using HSV as an amplicon vector. When comparing single-drug and multi-drug combination vaccinations, hepatectomy would benefit the latter (Delman et al., 2002). This information approaches the development of novel HSV-based immunotherapeutic strategies for the treatment of liver cancer. Cgal-Luc and H6-Luc, two oncolytic viruses created based on HSV-1, were genetically modified from HSV-1 17syn^+^ and HFEM, respectively. H6-Luc demonstrated unusual oncolytic activity of HCC cells (Argnani et al., 2011). In this research, it was also found that mice given intravenous or intrahepatic injections of both viruses experienced transient transduction, which was found using bioluminescent markers in multiple organs throughout the body. This finding may be related to the systemic immunity that oncolytic HSV exerts.

A liver cancer-specific lysing virus (LCSOV) was created to be used in HCC as a result of the miR-122 being discovered to be silenced in HCC cells. Studies showed that LCSOV is highly selective in its ability to reduce HCC xenografts and shows cytotoxicity against HCC cells (Fu et al., 2012). Another HSV-1-based oncolytic virus, Ld0-GFP, also demonstrated excellent killing capacity against HCC and may have promise for other tumor types (Luo et al., 2019). When aMPD-1 scFv is inserted into HSV-1, it becomes active for expression and allows for a continuous distribution of aMPD-1 scFv in the tumor microenvironment (TME). This improves TME, inhibits PD-1, and lowers tumor load in liver cancer models (Lin et al., 2020). The promising future of HSV as a vector for gene insertion, alteration, and expression to lyse tumor cells is still discernible from this study, in my opinion.

### 4.5 Gallbladder cancer and cholangiocarcinoma

Unfortunately, no current studies on HSV and gallbladder treatment are available, but studies on cholangiocarcinoma can demonstrate its potential developmental significance. Gallbladder cancer and cholangiocarcinoma are both types of biliary tract cancer; the only difference between the two is their location (Ethun et al., 2017; Banales et al., 2020). Cholangiocarcinoma is a malignant tumor that develops from the epithelial cells of the bile duct, whereas gallbladder cancer develops from the epithelial cells of the gallbladder wall. Although cholangiocarcinoma has a higher 5-year survival rate than gallbladder cancer (Banales et al., 2016; Gamboa and Maithel, 2020), both pose the same health risk.

Three human cholangiocarcinoma cell lines were employed for an experimental NV1023 mixed with radiation (XRT) treatment. NV1023 is an oncolytic HSV defective in γ_1_34.5. The outcomes demonstrated that although NV1023 had varying sensitivity to each of the 3 cells, they all strongly destroyed tumor cells, displaying cytotoxicity, and that the addition of radiation improved this result even further (Jarnagin et al., 2006). It is thought that radiation may enhance the tumor-lytic effect of NV1023 by upregulating growth arrest and DNA damage protein 34 (GADD34) expression since GADD34 is highly similar to γ_1_34.5 and promotes cell cycle arrest and DNA repair. However, various cell types must be separated. A new oncolytic HSV-1 known as VG161 carries genes for a number of anti-tumor immunomodulatory molecules, including IL-12, IL-15, and IL-15 receptor alpha subunit, as well as TF-Fc that prevents PD-1/PD-L1 interactions (Chouljenko et al., 2020). While maintaining patient safety, VG161 can simultaneously mediate the antitumor immune responses of several routes, exerting more cytotoxicity and better lysis. Intrahepatic cholangiocarcinoma (ICC) patient-derived xenograft (PDX) animals appear to exhibit improved antitumor activity in response to both HSV-1 preimmunization and multi-cycle VG161 therapy (Ding et al., 2022). This may provide support for a long-term VG161 treatment plan for individuals in clinical trials who are HSV-1 seropositive. It is suggested that more alluring HSVs be created and employed for the diagnosis or treatment of gallbladder cancer as well as other malignancies.

### 4.6 Colorectal cancer

According to the International Agency for Research on Cancer, colorectal cancer has become the third most common malignancy in 185 countries worldwide (Sung et al., 2021), and OV therapy research has become increasingly diverse, with the goal of benefiting patients.

HSV oncolytic therapy for colorectal cancer has been studied since before the turn of the century. The hrR3 vector derived from HSV-1, for example, has been shown to be effective in killing colon cancer cells both *in vivo* and *in vitro* (Yoon et al., 1998). Notably, hrR3 contains the herpes simplex virus thymidine kinase (HSV-TK) suicide gene, an HSV-1 product that converts GCV into the toxic metabolite triphosphate-GCV (GCV-TP) and increases cytotoxicity (Yi et al., 2018). As a result, using this approach in the field of gene-directed enzyme pre-drug therapy (GDEPT) for the treatment of colorectal cancer and other digestive system tumors is feasible (Zhou et al., 2016; Luo et al., 2018; Sawdon et al., 2021; Zhou et al., 2022). The HSV-TK gene must first be delivered to the site of the lesion, followed by the administration of GCV as a prodrug to malignant cells to generate the toxic activation response described above, which will rapidly kill tumor cells. This two-step approach was replaced by a one-step approach employing polymeric micellar nanocarriers that bind the gene and the prodrug simultaneously for simple and efficient delivery of the suicide gene and the prodrug, resulting in high toxicity in colorectal cancer HT-29 cells *in vitro* (Sawdon et al., 2021).

In a phase I clinical trial in 2009, groups of 12 patients with intrahepatic metastatic colorectal cancer received varying doses of NV1020 by hepatic arterial injection, which proved to be a reliable method of vascular delivery of HSV-1. With decreased levels of the tumor marker carcinoembryonic antigen (CEA), the phase I trial opened a new pathway for the administration of oncolytic drugs. One patient in the group that received the most injections of tumor lysate experienced a 25% decrease in tumor volume (Fong et al., 2009). Following targeted treatment, CEA levels continued to drop (Fong et al., 2009). However, a prior study found a small number of side effects in participants and the discovery of viruses in serum and saliva samples following NV1020 dosing (Kemeny et al., 2006). Despite the fact that this study’s findings indicated NV1020 can enter the hepatic artery safely, it is important to continue to monitor the situation and to undertake follow-up phase II and III clinical trials.

Not only that, but as we enter the twenty-first century, we see that combination therapy with HSV modification studies is becoming more common and popular, increasing the likelihood of multiple mechanisms of enhanced tumor lysis efficacy. In mouse lateral abdominal tumor models and *in vitro* colorectal cancer cells, loss of viral ribonucleotide reductase (RR) of HSV-1 mutant G207 in combination with IR resulted in significant tumor lysis enhancement, but no such synergistic effect was observed with combination therapy of parental virus with IR (Stanziale et al., 2002). This undoubtedly demonstrates that IR’s upregulation of RR activity function is the driving force behind this additional oncolytic effect. HSV1716Ing4, a novel variant of HSV1716 found to express growth inhibitor 4 (Ing4), had a oncolytic effect 1000-fold greater than HSV1716 in the study (Conner and Braidwood, 2012). With progeny output, Ing4 may contribute to HSV1716Ing4 infection of colorectal cancer cells. Its safety has also been established, with no replication found in any organ other than tumor tissue. Canerpaturev (C-REV) is the predecessor of HF10, and the combination of C-REV with cetuximab resulted in enhanced antitumor activity, allowing all three human colorectal cancer cell lines expressing different levels of epidermal growth factor receptor (EGFR) to exhibit a corresponding state of decay with the dose and time of C-REV administration. HSV-1 in combination with the chemotherapeutic agent bortezomib improves oncolytic efficiency in colorectal cancer cells, which was demonstrated by upregulating heat shock protein expression while dramatically inducing endoplasmic reticulum stress and activating the cystein-12 apoptotic pathway (El-Sayes et al., 2022). Furthermore, when combined with chemotherapeutic agents such as mitomycin C (mito), immune checkpoint therapy improves in colorectal cancer patients (El-Sayes et al., 2022).

It is obvious that HSV has the ability to treat cancers not only through its oncolytic action but also by opening up a lot of opportunities for the creation of additional treatment modalities. This modified oncolytic HSV can express high levels of IL-12 in infected cells, promote T-cell proliferation, and strongly express IFN-γ, playing a critical role in lysing cancer cells after interleukin 12 (IL-12) and C-X-C motif chemokine ligand 11 (CXCL11) are substituted for the HSV-1 ICP47 and ICP34.5 coding regions, respectively (Zhang et al., 2022a). The findings of a comparable study lend credence to this notion (Haghighi-Najafabadi et al., 2021). Hypoxia is a constant in the TME, which is attributed to the inefficient tumor vascular system’s poor oxygenation (Jing et al., 2019). The cytotoxicity of an HSV-1 (HSV-HMGB1) expressing the HMGB1 protein, which inhibits tumor cell aerobic respiration, was tested in both normoxic and hypoxic conditions against three colorectal cancer cell lines (HCT116, SW480, and HT29) and compared to the parental virus HSV-ble (Shayan et al., 2022). In both states, HSV- HMGB1 was significantly more virulent than its parental virus for HCT116 and SW480. However, under hypoxia, HT29 showed value-added promotion, indicating that HSV-HMGB1 is not applicable to all types of colorectal cancer. In the future, more broad-spectrum and effective HSVs for improving TME should be investigated.

## 5 Discussion and challenge

Oncolytic viruses are thought to be promising and play a significant part in innovative immunotherapies for the treatment of malignancies. A standout performance among the various new oncolytic viruses is oncolytic HSV. In recent decades, there has been uninterrupted research on oncolytic HSV. Its genomic advantage provides a platform for the insertion of many genes with anti-cancer potential. From this point of view alone, oncolytic HSV is bound to gain more momentum in the future as research continues to advance. In addition, pharmacological immunotherapies are not abandoned. We note that the use of immunotherapeutic agents in combination with oncolytic HSV is supported by wide-ranging clinical data (Bommareddy et al., 2018). The Japanese Ministry of Health approved G47Δ as a treatment for malignant glioma in 2021, in addition to the previously mentioned T-VEC approved by the FDA (Otani et al., 2022). To date, only a limited number of OV drugs have been approved, obviously due to thoughtful safety concerns. Not only the complex mechanism of action between OV and cancer cells matters, but also the mode of OV delivery needs to be carefully considered. Intratumoral injection of oncolytic HSV is probably the most efficient delivery method available (Jahan et al., 2021). We believe that more recent iterations of HSV-based lysing viruses will be used more frequently for the clinical treatment of tumors in the future, even though many of these viruses alter the editable region of HSV genes and are currently in clinical trials or are only in the experimental validation stage (Patel et al., 2016; Suzuki et al., 2021; Wang et al., 2022b; Liu et al., 2022; Tian et al., 2022).

For the treatment of digestive system malignancies, oncolytic HSV-1 is frequently mentioned and exhibits excellent therapeutic potential in a range of digestive system tumors (Hu et al., 2022; Tang et al., 2022). Oncolytic HSV-2, which mostly treats cancers of the non-digestive system, appears to have notably scant data within the same group. The vast majority of lysogenic HSVs are currently derived from lysogenic HSV-1, but one piece of evidence suggests that FusOn-H2 from HSV-2 has a far superior antitumor effect on a subpopulation of cancer cells than Baco-1 from HSV-1 (Fu et al., 2018). This is a source of concern. On the one hand, research on oncolytic viruses with HSV-2 origins is few, making it difficult to assess their benefits and drawbacks; on the other hand, it is possible that we overlooked viral vectors with superior effects.

Additionally, the percentage of HSV studies filled by various tumor forms falling under the general category of digestive system cancers varies greatly. There are not many examples, for instance, of oncolytic virus HSV for gallbladder cancer. This is where we would want to suggest the direction for future investigation because we found it troubling in our collection of reports. Many proactive attempts should be pursued in subsequent studies for the treatment of cancers such as gallbladder cancer, including *in vivo* and *in vitro* trials. However, we know that a large part of the reason that oncolytic HSV is effective in treating tumors lies in the ignition of a fire of systemic immune processes. The efficacy of the treatment is closely related to the degree of this burst of immunity. Therefore, we would like to emphasize here that the patient remains in a cheerful mood during the treatment, which can positively influence the immune inflammatory response in the body (Avvenuti et al., 2016) and contribute to the treatment of oncolytic HSV.

Undoubtedly, oncolytic HSV’s anticancer properties are valued. On the other hand, the concept of using HSV as a diagnostic tool based on its preferential replication in cancer cells is also welcomed (Kelly et al., 2016; Zhang et al., 2016). This is due to the fact that it is crucial for detecting the metastasis and spread of many malignancies, may be successful in preventing many secondary cancers, and has broad repercussions for disruption and therapy in the future. Therefore, further research must be done after the discovery of its diagnostic significance. Last but not least, additional clinical trials and analysis are required to confirm the effectiveness and safety of various oncolytic HSVs as tumor lysis and diagnostic agents using data from phase I, II, and III clinical trials. These studies will open up fresh possibilities for effective DST immunotherapy.
