# Supplementary material for: HSV: The scout and assault for digestive system tumors
Source: Front Mol Biosci. 2023 Feb 28;10:1142498. doi: 10.3389/fmolb.2023.1142498 (PMC10011716; doi:10.3389/fmolb.2023.1142498)
Supplement: Supplementary file 1 [file Table1.docx]

| **Name** | **Type** | **Genetic Modifications** | **Experiment Types** | **Target DSTs** | **Positive/Negative** | **References** |
| --- | --- | --- | --- | --- | --- | --- |
| G207 | HSV-1 | Deletion of two copies of γ_1_34.5, inactivation of UL39 | Clinical trials (NCT03911388, NCT04482933, NCT02457845, NCT00157703, NCT00028158); in vivo; in vitro | Gastric cancer; colorectal cancer | Positive | (Bennett et al., 2002;Stanziale et al., 2002) |
| G47Δ | HSV-1 | Deletion of α47 based on G207 | in vivo; in vitro | Esophageal cancer; gastric cancer | Positive | (Sugawara et al., 2020;Yajima et al., 2021) |
| NV1020 | HSV-1 | Deletion of UL56, one copy of ICP0, ICP4 and γ_1_34.5, insertion of a fragment of HSV-2 DNA encoding several glycoprotein genes | Clinical trials (NCT00149396, NCT00012155); in vivo; in vitro | Gastric cancer; colorectal cancer | Positive | (Bennett et al., 2002;Kemeny et al., 2006;Fong et al., 2009) |
| NV1066 | HSV-1 | Deletion of one copy of ICP0, ICP4 and γ_1_34.5, insertion of EGFP | In vivo; in vitro | Gastric cancer; pancreatic Cancer | Positive | (Eisenberg et al., 2010) |
| hrR3 | HSV-1 | Inactivation of UL39 | In vivo; in vitro | Colorectal cancer | Positive | (Yoon et al., 1998) |
| OH2 | HSV-2 | Deletion of ICP47 and two copies of γ_1_34.5, insertion of GM-CSF | Clinical trials (NCT05248789, NCT05232136, NCT05698459, NCT05235074, NCT04637698, NCT03866525, NCT04616443, NCT04386967, NCT05648006); in vivo | Esophageal cancer; gastric cancer; cholangiocarcinoma; colorectal cancer | Positive | (Zhang et al., 2021) |
| HF10 | HSV-1 | Deletion of UL43, UL49.5,  UL55, UL56 and LAT | Clinical trials (NCT03153085, NCT03259425, NCT02428036, NCT03252808, NCT02272855, NCT01017185); in vivo; in vitro | Pancreatic Cancer | Positive | (Kasuya et al., 2014;Yamamura et al., 2014) |
| Cgal-Luc | HSV-1 | Insertion of the luciferase gene based on HSV-1 17syn^+^ | In vivo; in vitro | Hepatocellular carcinoma | Positive | (Argnani et al., 2011) |
| H6-Luc | HSV-1 | Insertion of the luciferase gene based on HSV-1 HFEM | In vivo; in vitro | Hepatocellular carcinoma | Positive | (Argnani et al., 2011) |
| LCSOV | HSV-1 | Linking essential viral glycoprotein H gene with the liver-specific apolipoprotein E (apoE)-AAT promoter, insertion of complementary sequences from miR-122a, miR-124a, and let-7 | In vivo; in vitro | Hepatocellular carcinoma | Positive | (Fu et al., 2012) |
| Ld0-GFP | HSV-1 | Insertion of GFP based on an ICP0-Null HSV-1 | In vitro | Hepatocellular carcinoma | Positive | (Luo et al., 2019) |
| NV1023 | HSV-1 | Insertion of a fragment of HSV-2 DNA containing the genes US2-2 to US2-5 based on NV1020 | In vitro | cholangiocarcinoma | Positive | (Jarnagin et al., 2006) |
| VG161 | HSV-1 | Deletion of two copies of γ_1_34.5, insertion of IL-12, IL-15, IL15RA and PL-L1b (TF-Fc peptide) | Clinical trials (NCT05223816, NCT05223816, NCT04758897, NCT04806464); in vivo; in vitro | Colorectal cancer | Positive | (Chouljenko et al., 2020) |
| HSV1716Ing4 | HSV-1 | Deletion of two copies of γ_1_34.5, insertion of Ing4 | In vitro | Colorectal cancer | Positive | (Conner and Braidwood, 2012) |
| HSV-HMGB1 | HSV-1 | Deletion of two copies of γ_1_34.5, insertion of HMGB1 | In vitro | Colorectal cancer | Positive | (Shayan et al., 2022) |

Argnani, R., Marconi, P., Volpi, I., Bolanos, E., Carro, E., Ried, C., et al. (2011). Characterization of herpes simplex virus 1 strains as platforms for the development of oncolytic viruses against liver cancer. *Liver Int* 31**,** 1542-1553. doi:10.1111/j.1478-3231.2011.02628.x

Bennett, J.J., Delman, K.A., Burt, B.M., Mariotti, A., Malhotra, S., Zager, J., et al. (2002). Comparison of safety, delivery, and efficacy of two oncolytic herpes viruses (G207 and NV1020) for peritoneal cancer. *Cancer Gene Ther* 9**,** 935-945. doi:10.1038/sj.cgt.7700510

Chouljenko, D.V., Ding, J., Lee, I.F., Murad, Y.M., Bu, X., Liu, G., et al. (2020). Induction of Durable Antitumor Response by a Novel Oncolytic Herpesvirus Expressing Multiple Immunomodulatory Transgenes. *Biomedicines* 8**,** 484. doi:10.3390/biomedicines8110484

Conner, J., and Braidwood, L. (2012). Expression of inhibitor of growth 4 by HSV1716 improves oncolytic potency and enhances efficacy. *Cancer Gene Ther* 19**,** 499-507. doi:10.1038/cgt.2012.24

Eisenberg, D.P., Carpenter, S.G., Adusumilli, P.S., Chan, M.K., Hendershott, K.J., Yu, Z., et al. (2010). Hyperthermia potentiates oncolytic herpes viral killing of pancreatic cancer through a heat shock protein pathway. *Surgery* 148**,** 325-334. doi:10.1016/j.surg.2010.05.005

Fong, Y., Kim, T., Bhargava, A., Schwartz, L., Brown, K., Brody, L., et al. (2009). A herpes oncolytic virus can be delivered via the vasculature to produce biologic changes in human colorectal cancer. *Mol Ther* 17**,** 389-394. doi:10.1038/mt.2008.240

Fu, X., Rivera, A., Tao, L., De Geest, B., and Zhang, X. (2012). Construction of an oncolytic herpes simplex virus that precisely targets hepatocellular carcinoma cells. *Mol Ther* 20**,** 339-346. doi:10.1038/mt.2011.265

Jarnagin, W.R., Zager, J.S., Hezel, M., Stanziale, S.F., Adusumilli, P.S., Gonen, M., et al. (2006). Treatment of cholangiocarcinoma with oncolytic herpes simplex virus combined with external beam radiation therapy. *Cancer Gene Ther* 13**,** 326-334. doi:10.1038/sj.cgt.7700890

Kasuya, H., Kodera, Y., Nakao, A., Yamamura, K., Gewen, T., Zhiwen, W., et al. (2014). Phase I Dose-escalation Clinical Trial of HF10 Oncolytic Herpes Virus in 17 Japanese Patients with Advanced Cancer. *Hepatogastroenterology* 61**,** 599-605.

Kemeny, N., Brown, K., Covey, A., Kim, T., Bhargava, A., Brody, L., et al. (2006). Phase I, open-label, dose-escalating study of a genetically engineered herpes simplex virus, NV1020, in subjects with metastatic colorectal carcinoma to the liver. *Hum Gene Ther* 17**,** 1214-1224. doi:10.1089/hum.2006.17.1214

Luo, Y., Lin, C., Ren, W., Ju, F., Xu, Z., Liu, H., et al. (2019). Intravenous Injections of a Rationally Selected Oncolytic Herpes Virus as a Potent Virotherapy for Hepatocellular Carcinoma. *Mol Ther Oncolytics* 15**,** 153-165. doi:10.1016/j.omto.2019.09.004

Shayan, S., Arashkia, A., Bahramali, G., Abdoli, A., Nosrati, M.S.S., and Azadmanesh, K. (2022). Cell type-specific response of colon cancer tumor cell lines to oncolytic HSV-1 virotherapy in hypoxia. *Cancer Cell Int* 22**,** 164. doi:10.1186/s12935-022-02564-4

Stanziale, S.F., Petrowsky, H., Joe, J.K., Roberts, G.D., Zager, J.S., Gusani, N.J., et al. (2002). Ionizing radiation potentiates the antitumor efficacy of oncolytic herpes simplex virus G207 by upregulating ribonucleotide reductase. *Surgery* 132**,** 353-359. doi:10.1067/msy.2002.125715

Sugawara, K., Iwai, M., Yajima, S., Tanaka, M., Yanagihara, K., Seto, Y., et al. (2020). Efficacy of a Third-Generation Oncolytic Herpes Virus G47Δ in Advanced Stage Models of Human Gastric Cancer. *Mol Ther Oncolytics* 17**,** 205-215. doi:10.1016/j.omto.2020.03.022

Yajima, S., Sugawara, K., Iwai, M., Tanaka, M., Seto, Y., and Todo, T. (2021). Efficacy and safety of a third-generation oncolytic herpes virus G47Δ in models of human esophageal carcinoma. *Mol Ther Oncolytics* 23**,** 402-411. doi:10.1016/j.omto.2021.10.012

Yamamura, K., Kasuya, H., Sahin, T.T., Tan, G., Hotta, Y., Tsurumaru, N., et al. (2014). Combination treatment of human pancreatic cancer xenograft models with the epidermal growth factor receptor tyrosine kinase inhibitor erlotinib and oncolytic herpes simplex virus HF10. *Ann Surg Oncol* 21**,** 691-698. doi:10.1245/s10434-013-3329-3

Yoon, S.S., Carroll, N.M., Chiocca, E.A., and Tanabe, K.K. (1998). Cancer gene therapy using a replication-competent herpes simplex virus type 1 vector. *Ann Surg* 228**,** 366-374. doi:10.1097/00000658-199809000-00009

Zhang, B., Huang, J., Tang, J., Hu, S., Luo, S., Luo, Z., et al. (2021). Intratumoral OH2, an oncolytic herpes simplex virus 2, in patients with advanced solid tumors: a multicenter, phase I/II clinical trial. *J Immunother Cancer* 9. doi:10.1136/jitc-2020-002224
